# Drug-Induced Long-QT and *Torsades de Pointes* in Elderly Polymedicated Patients

**DOI:** 10.5935/abc.20150069

**Published:** 2016-02

**Authors:** Daniel García-Fuertes, Elena Villanueva-Fernández, Manuel Crespín-Crespín

**Affiliations:** Hospital Santa Bárbara, Puertollano, Ciudad Real - Spain

**Keywords:** Torsades de Pointes, Aged, Inappropriate Prescribing, Long QT Syndrome, Arrhythmias, Cardiac

## Introduction

Polymedication affects one in three patients older than 65 years.^1^ Its risks are widely known and are specially related to pharmacological interactions.^2^ One of these potential risks is the appearance of malignant ventricular arrhythmias when drugs that prolong QT interval are prescribed, including antibiotics, antidepressants, antiemetics, psychotropic medication or even antiarrhythmic drugs.^3-6^ The development of ventricular arrhythmias such as *Torsades de Pointes* (TdP), typically related to QT prolongation, is a potentially lethal complication. It is mandatory to recognize the drugs that can cause it, avoiding their joint use or planning a close monitoring in case their combination cannot be avoided.

## Case Report

Three consecutive cases of polymedicated patients presenting with polymorphic ventricular tachycardia due to a pharmacological induced prolonged QT interval are presented.

### Patient 1

An 84 year-old woman was admitted to the emergency department because of syncope. The patient had a history of hypertension, dyslipidemia, permanent atrial fibrillation, anxiety-depressive disorder and aortic and mitral valve replacement with residual moderate left ventricular dysfunction. She was receiving treatment with acenocoumarol, furosemide, candesartan, digoxin, simvastatin, sulpiride and escitalopram. She had also recently initiated treatment with solifenacin due to urinary incontinence.

**Figure 1:**
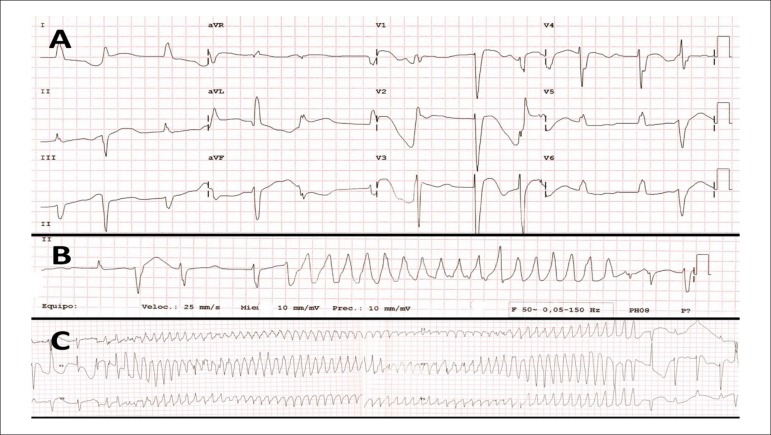
Twelve-lead electrocardiogram of the patient in case 1. A. Atrial fibrillation with controlled ventricular rate, complete left bundle-branch block, prolonged corrected QT interval and frequent premature ventricular complexes. B. Non-sustained wide QRS-complex tachycardia. C. Torsades de Pointes.

Her 12-lead electrocardiogram (ECG) showed atrial fibrillation with a controlled ventricular rate, previously known complete left bundle-branch block, a prolonged corrected QT interval (558 ms, Hodges method), frequent premature ventricular complexes (Figure 1A) and episodes of wide QRS-complex tachycardia (Figure 1B and 1C) compatible with TdP. Laboratory tests revealed hypokalemia (3.4 mEq/L) and hypomagnesemia (1.67 mg/dL).

Initially, intravenous amiodarone and magnesium sulfate were administered. Levofloxacin was also initiated due to respiratory infection symptoms. Sustained and non-sustained polymorphic ventricular tachycardia persisted and multiple electrical shocks were delivered to the patient due to hemodynamic instability. Once the cardiologist evaluated the patient, all drugs prolonging QT interval were withdrawn, hydroelectrolytic disturbances were corrected and temporary transjugular ventricular pacing at 90 b.p.m. was performed. The patient did not have any new arrhythmic events, ventricular pacing was stopped at 48 hours and QT interval progressively normalized.

### Patient 2

An 85 year-old, diabetic and hypertensive woman was hospitalized due to complicated biliary colic. She had history of paroxysmal atrial fibrillation, hypertensive cardiomyopathy, depression and vertiginous syndrome. Her treatment included: losartan, betahistine, simvastatin, amiodarone, bisoprolol, acenocoumarol, metformin, iron sulfate and escitalopram. She had also been treated with metoclopramide due to of nausea and vomiting.

On the fifth day of hospital stay she underwent cardiorespiratory arrest. Basic cardiopulmonary resuscitation was initiated and a self-limited TdP was identified when the ECG was monitored. Her 12-lead ECG showed sinus rhythm, left bundle-branch block and a prolonged corrected QT interval (475 ms, Hodges method). Hypokalemia (3.3 mEq/L) and hypomagnesemia (1.5mg/dL) were also found. QT-prolonging drugs were withdrawn and electrolytic disturbances were corrected by means of intravenous potassium and magnesium sulfate. No new events occurred.

### Patient 3

A 74 year-old, diabetic, hypertensive, dyslipidemic and active smoker woman was admitted because of heart failure. She had a history of atrial fibrillation and rheumatic valve disease with mild mitral stenosis and regurgitation, moderate aortic regurgitation and severe tricuspid regurgitation. She was also diagnosed with severe chronic obstructive pulmonary disease and moderate cognitive impairment. Her treatment included acenocoumarol, bisoprolol, simvastatin, indapamide, paroxetine, sulpiride, omeprazole, paracetamol, tramadol, risedronic acid, alprazolam and metoclopramide.

Her ECG showed atrial fibrillation with rapid ventricular rate, frequent premature ventricular complexes and a prolonged corrected QT interval (565 ms, Bazzet method). A few hours after admission she underwent cardiorespiratory arrest with multiple episodes of TdP that degenerated into ventricular fibrillation. Blood tests showed hypomagnesemia (1.34 mg/dL), hypocalcemia (8.5 mg/dL) and kalemia of 3.6 mmol/L. Multiple electrical shocks were delivered. Prior to cardiologic evaluation intravenous magnesium sulfate and amiodarone were administered, in addition to electrolytic disturbance correction. After cardiologic evaluation, amiodarone and other QT-prolonging drugs were withdrawn. The patient became asymptomatic, with no further episodes of ventricular arrhythmias and normalization of the corrected QT interval.

The patients remained asymptomatic in relation to ventricular arrhythmias at a mean follow-up of seven months after hospital discharge (11, 3 and 7 months for each patient, respectively). Table 1 summarizes the main clinical features of the 3 patients, number of chronically prescribed drugs, QT-prolonging drugs, hydroelectrolytic disorders favoring QT prolongation and definitive treatment in each case. The Hodges method was used for the correction of the QT interval measurement in the presence of left bundle-branch block, as its results are more reliable than those obtained with Bazzet formula.^7^

**Table 1:** Clinical features and treatment of the patients

	**Case 1**	**Case 2**	**Case 3**
**Gender**	**Female**	**Female**	**Female**
Age	84	85	74
Number of chronic drugs	8	9	10
Prolonging-QT drugs	Amiodarone		
	Escitalopram	Amiodarone	Indapamide
	Levofloxacin	Escitalopram	Metoclopramide
	Solifenacin	Metoclopramide	Paroxetine
	Sulpiride		Sulpiride
Hypokalemia	+	+	-
Hypomagnesemia	+	+	+
Hypocalcemia	-	-	+
Heart disease	+	+	+
Torsades de Pointes	+	+	+
Ventricular Fibrillation	-	-	+
**Treatment**			
Drug withdrawal	+	+	+
Magnesium sulfate	+	+	+
Potassium	+	+	+
Isoproterenol	-	-	-
Pacemaker	+	-	-

+: clinical feature was present or treatment was administered; -: clinical feature was not present or treatment was not administered.

## Discussion

Polymedication in elderly patients may carry higher risk of severe adverse events, especially when QT-prolonging drugs are co-administered. QT-prolonging drugs can be categorized by their potential to cause QT prolongation and/or TdP into: drugs with known risk of TdP (amiodarone, escitalopram, levofloxacin, sulpiride), drugs with possible risk of TdP and drugs with conditional risk of TdP (indapamide, paroxetine, solifenacin). All of our patients were treated with at least one drug categorized as "known risk of TdP". Furthermore, two of the three described patients received additional prolonging-QT drugs even after documentation of QT prolongation and polymorphic ventricular tachycardia (amiodarone and levofloxacin in case 1, and amiodarone in case 3).

Electrolytic disturbances contributing to TdP were also found in all cases (hypokalemia, hypomagnesemia and/or hypocalcemia). The three cases were reported in elderly women with structural heart disease. All these factors have been described as risk factors for TdP.^8,9^

It is noteworthy that amiodarone was used as a first line treatment in two of the three cases. It is common practice to use amiodarone in the setting of a ventricular tachycardia. Despite its usefulness in the treatment of monomorphic ventricular tachycardia, it is contraindicated in polymorphic ventricular tachycardia such as TdP due to a prolonged QT interval. According to clinical practice guidelines, treatment should include withdrawal of any offending drugs, correction of electrolyte abnormalities (potassium repletion to 4.5 to 5 mmol/L may be considered) and intravenous magnesium sulfate. High rate pacing is reasonable for patients who present with recurrent pause-dependent *torsades de pointes*, usually due to premature ventricular complexes with a short-long-short sequence, as it happened in case 1. Isoproterenol can be used alternatively.^10^

Special effort is being made by scientific societies in order to reduce potentially inappropriate medications, which continue to be prescribed and used as first-line treatment for the most vulnerable of older adults, despite evidence of poor outcomes. Some of the drugs prescribed to our patients, such as amiodarone or digoxin, are considered inappropriate medications according to the American Geriatrics Society Updated Beers Criteria for Potentially Inappropriate medication Use in Older Adults^11^ and should be avoided in older adults.

## Conclusions

Polymedication involves a high risk of adverse effects. It Therefore, it is crucial to identify patients receiving drugs that can induce QT interval prolongation and perform serial electrocardiograms, due to the potential risk of ventricular arrhythmias.
